# Prevalence of vaccine-derived hepatitis B surface antibodies in children and adolescents in Germany: results from a population-based survey, 2014–2017

**DOI:** 10.1186/s12879-024-09201-7

**Published:** 2024-03-15

**Authors:** Ida Sperle, Sofie Gillesberg Lassen, Martin Schlaud, Achim Dörre, Sandra Dudareva, Christina Poethko-Müller, Thomas Harder

**Affiliations:** 1 Department of Infectious Disease Epidemiology, Robert Koch Institute, Berlin, Germany; 2 Postgraduate Training for Applied Epidemiology (PAE), Robert Koch Institute, Berlin, Germany; 3 ECDC Fellowship Programme, Field Epidemiology Path (EPIET), European Centre for Disease Prevention and Control (ECDC), Stockholm, Sweden; 4 PhD Programme, Charité – Universitätsmedizin Berlin, Berlin, Germany; 5 Department of Epidemiology and Health Monitoring, Robert Koch Institute, Berlin, Germany

**Keywords:** Hepatitis B, Vaccination, Germany, Anti-HBs level

## Abstract

**Introduction:**

Childhood vaccination against hepatitis B has been recommended in Germany since 1995. WHO defines a primary vaccination series as successful if the initial hepatitis B surface antibody (anti-HBs) level is ≥ 10 IU/L directly after vaccination. Anti-HBs levels vary depending on the number of doses, type of vaccine, and time interval between the last two doses. In 2021, Germany began to recommend three instead of four doses of polyvalent hepatitis-B-containing vaccines. Our aim was to estimate the proportion of vaccinated children in Germany with anti-HBs levels < 10 IU/L, 10–99 IU/L, and ≥ 100 IU/L by number and type of vaccine, and assess if number of doses and compliance with recommended time interval between the last two doses are associated with an anti-HBs level ≥ 10 IU/L when considering type of vaccine and time since last dose.

**Methods:**

We used data from a national cross-sectional study (2014–2017) of children (3–17 years). We excluded participants with unknown vaccination dates, unreadable or incomplete vaccination cards, and hepatitis B virus (HBV)-positive participants. We defined a recommended schedule as a vaccination series with at least six months between the two last doses and having three doses or more. We calculated weighted anti-HBs sero-prevalence for three anti-HBs levels: < 10 IU/L, 10–99 IU/L and ≥ 100 IU/L. We fitted two logistic regression models to examine the relationship between number of doses and recommended schedule on anti-HBs levels (≥ 10 IU/L and ≥ 100 IU/L) considering time since last dose and type of vaccine (Infanrix, Hexavac, Monovalent).

**Results:**

We included 2,489 participants. The weighted proportion of vaccinated children per anti-HBs level was < 10 IU/L: 36.3% [95%CI 34.0–38.7%], 10–99 IU/L: 35.7% [33.2–38.2%] and ≥ 100 IU/L: 28.0% [25.9–30.2%]. We did not find an association between a recommended schedule of three versus four doses and anti-HBs ≥ 10 IU/L or ≥ 100 IU/L.

**Conclusions:**

Anti-HBs levels in later childhood were about equal, whether children received three or four doses. This implies that the change in the recommendations does not affect the anti–HBs level among children in Germany. Future studies are needed on the association of anti-HBs levels and adequate sustained protection against HBV.

**Supplementary Information:**

The online version contains supplementary material available at 10.1186/s12879-024-09201-7.

## Introduction

Viral hepatitis B is a serious public health challenge, and a leading cause of acute and chronic liver disease globally [1, 2]. Chronic hepatitis B can lead to severe long-term sequelae such as liver cirrhosis and hepatocellular carcinoma [1]. Hepatitis B virus (HBV) infections acquired through mother-to-child transmission are often asymptomatic, and more than 90% of those infected during infancy and early childhood develop a chronic infection compared to only 5% of people who are infected as an adult [1, 3].

World Health Organization (WHO) estimates that 14 million people are living with chronic HBV infection (2019) [4], and long-term sequelae caused by chronic HBV infections are responsible for about 56,000 deaths per year in the WHO European Region [5]. In Germany, an HBV prevalence (antibody to hepatitis B core antigen (anti-HBc) and hepatitis B surface antigen (HBsAg)) of 0.3% has been found in the latest adult population-based survey (German Health Interview and Examination Survey for Adults (DEGS1, 2008–2011)) [6]. Among children aged three to 17 years in Germany, the prevalence of the surface antigen of HBV (HBsAg, current infection) was 0.2% in 2003–2006 [6, 7].

In 2016, WHO published the first global health sector strategy on viral hepatitis, offering a plan that included specific impact targets and goals to eliminate viral hepatitis as a public health problem by 2030. Childhood vaccinations are a central intervention for hepatitis B elimination [8]. The action plan for ending viral hepatitis in the WHO European Region included the target of reaching an overall coverage of 95% of the population having three doses of the HBV vaccine by 2020 to reach HBV elimination by 2030 [9, 10]. Evidence from Germany indicates that the childhood vaccination coverage is below this threshold [11].

While treatment of chronic hepatitis B infection may lead to seroconversion, there is no cure [1]. A life-long treatment is usually required to maintain a low viral load and prevent the development of long-term sequelae [1]. This, as well as the high risk of chronic infection if infected early in life, emphasizes the importance of primary prevention of hepatitis B infection during childhood.

An effective and safe vaccine has existed since 1982, and currently available vaccines offer a high protection against infection with hepatitis B [12–14]. In Germany, vaccination against HBV has been recommended to all infants, children and adolescents since 1995 in the national immunisation schedule, as outlined by the German Standing Committee on Vaccination (STIKO) [15].

Ensuring effective protection following hepatitis B vaccination has important implications for both individuals and public health. While vaccination offers good protection and has proved to be an important intervention to reduce transmission, the exact duration of hepatitis B protection after childhood immunisation remains unclear. Some studies suggest several years or even lifelong protection [12, 16–20], while others indicate 10–15 years [18, 21]. A study from Germany demonstrated lower anti-HBs levels after HBV childhood immunisation and reduced immunogenicity when given the polyvalent vaccine Hexavac [22], which was withdrawn from the market in 2012 [23].

Successful primary vaccination has been defined as when the individuals have anti-HBs levels ≥ 100 IU/L 4–6 weeks after vaccination by STIKO [24], whereas WHO defines an initial anti-HBs level of ≥ 10 IU/L as indication of successful vaccination [25]. However, the anti-HBs level decreases over time and furthermore does not necessarily reflect immunity [20, 26, 27].

Studies also suggest that there is a difference in anti-HBs levels after primary vaccination according to the number of doses given (three or four) and the time interval between the last two vaccine doses [28, 19]. However, important open questions about the duration of hepatitis B protection following childhood immunisation remain. One of these include whether the recommended six months between the two last doses impacts the stability of the anti-HBs level over time [29].

Until recently, either four doses of polyvalent vaccine or three doses of monovalent vaccine were recommended for infant vaccination in Germany. In June 2021, the recommendations were changed to three doses which should be provided at two, four and 11 months of age with a minimum of six months between the last two doses, regardless of vaccine type. Only children born to a mother with unknown HBV status or who are infected with HBV are offered a birth-dose simultaneously with immunoglobulin [30].

A study by Gillesberg Raiser et al. [31] found that 91.4% (95%CI: 89.7%–92.8%) of children and adolescents in Germany who did not receive a birth-dose, received three or more doses of hepatitis B vaccination. However, only 79.1% (95%CI: 76.8%–81.2%) received the last dose with at least six months between the two last doses as recommended.

We aimed to estimate the proportion of hepatitis B vaccinated children and adolescents (three to 17 years) in Germany with an anti-HBs level < 10 IU/L, 10–99 IU/L and ≥ 100 IU/L. Moreover, we wanted to assess if three versus four doses and compliance with the recommended six months interval between the last two doses are associated with an anti-HBs level ≥ 10 IU/L when taking type of vaccine and time since the last dose into account.

## Methods

We used data from the second wave of the German Health Interview and Examination Survey for Children and Adolescents (KiGGS Wave 2), a nationwide population-based cross-sectional study conducted from 2014–2017 [32].

KiGGS Wave 2 is comprised of two components each with its own sampling procedure: interviews only and interviews combined with physical health examinations. A detailed description of the methodology of KiGGS and KiGGS Wave 2 is published elsewhere [32, 33]. In brief, an age-stratified population sample was drawn of three to 17-year-olds who were invited to take part in the physical health examination component. A total of 3,567 children and adolescents participated in the examination component, which corresponds to a response rate of 41.5%.

Data on sociodemographic factors and general health status through a large number of health indicators were collected using questionnaires and interviews [32]. The socio-economic status is measured through the collection of education and professional qualification, employment status of parents and net household income which are converted into a seven point index scale. This original index scale was then categorised into five groups [34]. Information on date, number and type of vaccinations was collected from vaccination cards.

**Fig. 1:**
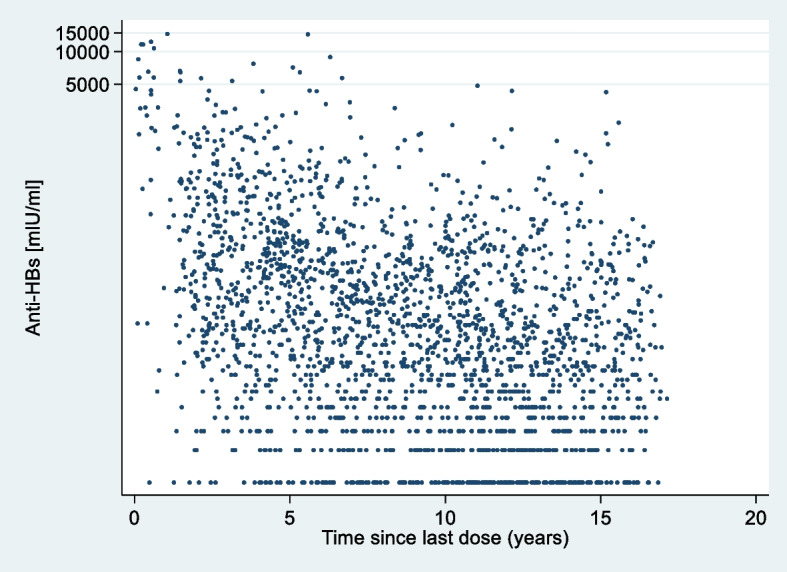
Time since last dose (in years) and anti-HBs level (*N* = 2,472*). *17 samples had anti-HBs values above the upper detection limit and were excluded from Figure 1

Blood samples were collected from participants as part of the physical health examinations. The blood samples were analysed for antibodies against hepatitis B (anti-HBs and anti-HBc) and HBV surface antigen (HBsAg) using the Abbott Laboratories (Illinois, USA) Architect system. The anti-HBs assay used had an overall specificity of 99.67% (95% CI 99.2%–99.9%) and a sensitivity of 97.5% (95% CI 95.9%–98.6%) with a limit of blank of 0,43 IU/L, a limit of detection of 0,90 IU/L, and a range of quantitation of 2,50–1000,00 IU/L. Serum specimens with a level of anti-HBs exceeding 1000 IU/L were automatically diluted and re-analysed. Diluted samples still exceeding the upper range of quantitation (now 25,000 IU/L) were not further processed and excluded from Fig. 1, but included in the other quantitative data analyses (*n* = 17) [35].

We excluded participants with unknown vaccination date, or with unreadable or incomplete information on hepatitis B vaccination. Furthermore, we excluded participants with missing anti-HBs measurements, or with signs of previous or current HBV infection (anti-HBc or HBsAg positive participants).

We defined a recommended schedule as one with three or more vaccine doses with at least six months between the last two doses.

### Statistical analysis

Our descriptive analysis of the study population included relevant variables for socio-demographic aspects (age, sex, geographical place of living, migration status), vaccination (time since last dose, type of vaccine) and anti-HBs titre. We derived frequencies for categorical variables, and determined interquartile ranges (IQR) for relevant variables, including those used for weighting. A weighting factor was created for KiGGS Wave 2 to ensure that the cross-sectional component was nationally representative regarding age, sex, parental level of education and nationality (“German: Yes/No”). Additionally, it accounts for the difference in probability of participation and also adjusts for deviations if the design-weighted net sample from the German population [32].

We used chi-squared tests to compare sex and geographical place of living (East or West Germany), and Student’s unpaired *t*-test to compare the mean age among included and excluded participants.

We categorised the anti-HBs level in three groups: < 10 IU/L, 10–99 IU/L and ≥ 100 IU/L. We estimated proportions with 95% confidence intervals (CI) weighted by age group, sex and recommended schedule with either three or four doses. The weighted proportions were calculated in order to account for unequal probabilities of selection for the sample, including differences in sex, age, geographical area (federal states), education (parental) and migration status [32]. We examined the distribution of the anti-HBs level and time since last dose (in years), stratified by sex and age group. We conducted linear regression analysis to examine the association between time since last dose in years and anti-HBs level as a continuous variable, considering sex, recommended schedule, and three versus four doses. We selected independent variables to be included in the analysis based on previous literature: time since vaccination and type of vaccine [19, 22, 28]. For type of vaccine, we conducted three analyses; one where monovalent vaccinations were included as a third category as presented in the results (Monovalent, Hexavac, Infanrix Hexa), one where monovalent vaccinations were excluded (Hexavac, Infanrix Hexa) and one where only participants with Infanrix Hexa were included. A participant who had received at least one dose of Hexavac was categorised as having a vaccination series with Hexavac.

To investigate which variables are associated with the anti-HBs level, we considered two logistic regression models for two binary outcome variables: anti-HBs ≥ 10 IU/L (yes/no) and anti-HBs level ≥ 100 IU/L (yes/no), and recommended schedule versus non-recommended schedule (Model 1) and three versus four doses (Model 2) as independent variables, respectively. Additional independent variables included in the model were identified through literature [22, 28, 19]. We performed all statistical analyses and created the figures in STATA™ (software version 17.0, StataCorp).

## Results

Three thousand five hundred sixty-seven children and adolescents participated in the examination part of KiGGS Wave 2. Of them, 402 participants were excluded due to not having their vaccination card, or it being illegible, and another 206 due to not having any HBV vaccination records. Six participants had signs of current or previous HBV infection and 464 had no anti-HBs titre measurements (Additional file 1). Finally, 2,489 were included in our analyses.

Of the 2,489 included participants, 50.7% were female. The median age was 11 years (IQR: 7–14). Nearly two thirds of the participants lived in West Germany (64.1%). 21.5% had a migration background (at least one parent). 76.4% of participants had received a polyvalent HBV vaccination series (Table 1).

**Table 1:** Distribution of socio-demographic and health characteristics of the study population, *N* = 2,489

**Characteristics**	***n* (%)**
**Sex**	
Male	1,226 (49)
Female	1,263 (51)
**Age group (years)**	
3–6	525 (21)
7–10	613 (25)
11–13	619 (25)
14–17	732 (29)
**Geographical place of living**	
Eastern Germany (incl. Berlin)	893 (36)
WesternGermany	1,596 (64)
**Socio-economic status**	
Low (1st Quintile)	357 (14)
Middle (2nd Quintile)	483 (19)
Middle (3rd Quintile)	465 (19)
Middle (4th Quintile)	546 (22)
High (5th Quintile)	581 (23)
Missing	57 (2.3)
**Migration status**	
None	1,908 (77)
One-sided (one parent born abroad or no German citizenship)	212 (8.5)
Two-sided (both parents born abroad or no German citizenship)^a^	324 (13)
Missing	45 (1.8)
**Type of vaccine**	
Monovalent	588 (24)
Polyvalent^b^:	1,901 (76)
Hexavac	409 (16)
Infanrix Hexa	1,401 (56)

^a^Migration status is also considered two-sided if the child and at least one parent are born abroad or do not have German citizenship

^b^91 participants received a polyvalent vaccine but the type, whether Hexavac or Infanrix Hexa, was unknown

KiGGS Wave 2 participants included in our analysis differed slightly from those excluded with regard to age and place of residence. The median age of the included participants (*N* = 2,489) was 11 years, compared to 9 years for the 1,078 excluded participants (*p* < 0.001). The proportion of participants living in East Germany (including Berlin) was slightly higher for included participants (35.9%) than excluded participants (31.2%) (*p* < 0.001) (Additional file 2).

### Anti-HBs response according to demographic characteristics and time since last dose

The mean time since the last vaccination dose for all participants was 3245.5 days (SD = 1552.8), corresponding to 8.9 calendar years. The distribution of the anti-HBs level versus time since the last dose is shown in Fig. 1.

The distribution was similar (Additional file 3) for the anti-HBs level versus time since last dose, when stratified by sex and age group.

More than one third of vaccinated children and adolescents had anti-HBs levels below the threshold of 10 IU/L (36.3% [95% CI 34.0–38.7%]). There was a slightly higher proportion of females than males with anti-HBs levels < 10 IU/L. For age groups, a higher anti-HBs level in the three to six years old age group. Moreover, there was a higher proportion of participants with anti-HBs levels < 10 IU/L, 10–99 IU/L and ≥ 100 IU/L compared to Hexavac and monovalent vaccinations (Table 2).

**Table 2:** Mean time since last dose, and weighted proportion of anti-HBs seroprevalence by sex, age and type of vaccine at time of examination (*N* = 2,489)

	**Total included participants**	**Time since last dose (years, mean)**	**Anti-HBs level < 10 IU/L**		**Anti-HBs level 10–99 IU/L**		**Anti-HBs level ≥ 100 IU/L**	
	**n**		**n**	**%**	**n**	**%**	**n**	**%**
Total	2,489	8.9	959	36 [34–39]	900	36 [33–38]	630	28 [26–30]
**Sex**								
Female	1,263	9.1	527	40 [36–43]	436	34 [30–37]	300	27 [24–30]
Male	1,226	8.7	432	33 [30–37]	464	38 [34–41]	330	29 [26–33]
**Age**								
Age 3–6	525	3.6	89	15 [12–19]	199	38 [32–43]	237	47 [42–53]
Age 7–10	613	7.3	223	38 [33–43]	256	40 [35–45]	134	22 [18–26]
Age 11–13	619	10.5	313	48 [42–53]	203	34 [28–39]	103	19 [15–24]
Age 14–17	732	12.6	334	46 [41–51]	242	32 [27–37]	156	22 [19–26]
**Type of vaccine**								
Monovalent	588	11.8	243	42 [37–47]	204	32.9 [28–38]	141	25 [22–30]
Polyvalent^a^:	1,901	8.0	716	35 [32–37]	696	37 [34–40]	489	29 [26–31]
Hexavac	409	11.2	270	62 [55–68]	111	31 [25–37]	28	8 [5–13]
Infanrix Hexa	1,401	7.1	415	28 [25–31]	564	39 [35–42]	422	34 [31–37]

^a^91 participants received a polyvalent vaccine but the type, whether Hexavac or Infanrix Hexa, was unknown

### Anti-HBs level by number of doses and type of HBV vaccination

When stratifying by recommended schedule, the estimated proportions of participants with levels of anti-HBs ≥ 10 IU/L were comparable for whether participants were vaccinated on the recommended schedule, or not. However, a higher proportion of participants with four doses had anti-HBs ≥ 10 IU/L than participants with three doses (Table 3).

**Table 3:** Anti-HBs level by number and type of vaccination at time of examination, weighted proportions (*N* = 2,489)

	**Total *n***	**Anti-HBs level < 10 IU/L**		**Anti-HBs level 10–99 IU/L**		**Anti-HBs level ≥ 100 IU/L**	
		n	% [95%CI]	n	% [95%CI]	n	% [95%CI]
**Recommended schedule** (≥ 3 doses)							
Yes	2,130	818	35.8 [33.2–38.4]	764	35.6 [32.8–38.5]	548	28.6 [24.8–29.1]
No	359	141	39.7 [33.7–45.9]	136	36.0 [30.1–42.3]	82	24.4 [19.7–29.7]
**Recommended schedule**							
3 doses	454	201	45.1 [39.4–51.0]	147	31.5 [26.3–37.1]	106	23.5 [18.8–29.0]
4 doses	1,644	610	33.5 [31.0–36.5]	610	37.1 [33.9–40.5]	424	29.4 [26.5–32.3]

In a linear regression analysis considering time since last dose, we found decreasing anti-HBs concentrations with each year that passed since the last dose (−51.8; 95% CI −60.04 to −43.60). When taking sex, recommended schedule, and three versus four doses into account, the trend remained the same (−58.1; 95% CI −67.32 to −48.92).

In univariable logistic regression analyses, we did not find a statistically significant association between having a recommended schedule and anti-HBs level ≥ 10 IU/L in later life (crude OR 1.2 [95% CI 0.89–1.57]). Having four rather than three doses (reference category) was positively associated with having an anti-HBs level ≥ 10 IU/L later on (crude OR 1.6 [95% CI 1.2–2.1]).

In the multivariable logistic regression models, neither having been vaccinated by the recommended schedule nor having four doses was found to have odds ratios different from 1 for both anti-HBs level ≥ 10 IU/L and anti-HBs level ≥ 100 IU/L when accounting for time since the last dose and type of vaccine (Table 4).

**Table 4:** Factors associated with a positive anti-HBs level – results of multivariable models (*N* = 2,489)

	**Anti-HBs level ≥ 10 IU/L**		**Anti-HBs level ≥ 100 IU/L**	
	**OR**	**95% CI**	**OR**	**95% CI**
**Model 1** (Recommended schedule (≥ 3 doses))				
Recommended schedule (≥ 3 doses)				
Yes	0.92	0.65–1.32	1.16	0.79–1.70
No	Reference		Reference	
Time since last vaccination dose (in years)	0.85	0.82–0.88	0.83	0.79–0.86
Type of vaccine				
Infanrix Hexa	Reference		Reference	
Hexavac	0.44	0.30–0.64	0.38	0.21–0.72
Monovalent	1.38	0.98–1.96	2.06	1.42–2.99
**Model 2; 3 versus 4 doses** (Recommended schedule (≥ 3 doses))				
3 versus 4 doses				
3 doses	Reference		Reference	
4 doses	1.44	0.81–2.60	1.06	0.60–1.85
Time since last vaccination dose (in years)	0.85	0.82–0.89	0.83	0.79–0.86
Type of vaccine				
Infanrix Hexa	Reference		Reference	
Hexavac	0.47	0.31–0.69	0.42	0.20–0.85
Monovalent	1.95	1.06–3.57	2.11	1.23–3.63

For both anti-HBs ≥ 10 IU/L and ≥ 100 IU/L, the time since the last dose and the type of vaccine had a significant association with the anti-HBs level. Compared to Infanrix Hexa (reference), having received Hexavac decreased the odds of having an anti-HBs level of > 10 IU/L or > 100 IU/L (OR = 0.44 [95% CI 0.30–0.64] and OR = 0.38 [95% CI 0.21–0.72]). Participants who received monovalent vaccinations had a greater odds of having an anti-HBs level of > 100 IU/L (OR = 2.06 [95% CI 1.42–2.99]) than those who received Infanrix Hexa.

The multivariable models (Table 4) used various categorisations of the type of vaccine, and yielded similar relationships between anti-HBs levels and the time since vaccination, or the type of vaccine (Model 1) and time since vaccination (Model 2). We did not find any statistically significant relationship between the anti-HBs level and the number of doses (Additional file 4).

## Discussion

In our study, we did not find that participants who had been vaccinated on the recommended schedule, or who had four doses of HBV vaccine, had higher levels of anti-HBs when taking time since last dose and type of vaccine into account, which is a result that has previously been reported in the literature [19, 22, 28]. There are several possible explanations for this. First, it is important to underline that having been vaccinated on the recommended schedule or having received four doses may have an impact on immune persistency and immunological memory, and individuals with low and decreasing anti-HBs levels may still be protected [22]. It is also likely that the impact of time since last dose (especially since antibody levels are measured at study participation) and type of vaccine is so strong that any associations with other variables are difficult to detect. Furthermore, when looking at the categories of three versus four doses and recommended versus not recommended schedule, there are only a few participants in the non-recommended schedule group and the group with three doses, especially in the anti-HBs ≥ 100 IU/L category. We found time since last dose and type of vaccine to be independently associated with anti-HBs concentration which is consistent with what has been found in other studies [22, 19, 27–29, 36].

Our results confirmed the importance of time since last dose, and the longer the time since the last dose, the lower the anti-HBs concentration. While anti-HBs level does not equal protection, looking at time in relation to anti-HBs concentration is important as the purpose of the childhood HBV vaccine programme is to ensure protection against HBV infection throughout childhood and adolescence. Infection early in life is associated with a much larger risk of chronic infection and sequalae, and therefore reducing risk of infection in early life is important for individuals and public health. For this study, we did not have a longer follow-up time to assess whether protection may last until adulthood, which would be needed to further answer the question of the duration of HBV protection and how it depends on the level of anti-HBs.

Biological sex has been reported in the literature to have an influence on anti-HBs titre, with females in general having a better post-vaccination response [37–40]. The differences in immune response is caused by both genes and hormones, and the immune response changes in males and females over time [41]. In our study we did not include biological sex as an individual variable in our models. Among the KiGGS participants, a higher proportion of males have received a recommended schedule in the older age groups. We tested the effect of biological sex in the multivariable models and sex did not impact the effect and association between our variables of interest and anti-HBs concentration. More than half of the participants however were also younger than 11 years of age, and therefore the effect of hormones and females reaching puberty may not be detectable among the participants in our study [41].

Our results confirmed previous findings that Hexavac has a weaker immunogenicity than Infanrix Hexa, illustrated in our data as being negatively associated with anti-HBs level ≥ 10 IU/L [22]. While in some individuals the anti-HBs concentration decreased to a lower or undetectable level, immunity may remain due to HBsAg-specific immunological memory [22]. This biological response may be linked to factors which we were unable to assess with the available data.

Thus far, the WHO and STIKO in Germany do not recommend booster vaccinations after completion of the three-dose vaccination programme [12, 30]. However, considering that more than one third of the children and adolescents in our study had an anti-HBs level under 10 IU/L, further information on duration and actual protection would help answer whether a booster vaccination would be called for to maintain immunity. One systematic review found that the protection varies, and that based on the anti-HBs titre (e.g. less than 10 IU/L), a booster dose should be administered [29]. However, the study also stresses the lack of data in particular from large sero-prevalence studies among adults that have been vaccinated against HBV as part of the childhood vaccination programme. Another larger study from Taiwan, which consisted of a series of cross-sectional serological surveys of HBV markers in four age groups between 2004 and 2012 found that booster vaccination did not add protection against HBsAg [42]. A study which measured residual immunity 10–16 years after vaccination in Canadian children [43] found that despite absence of HBV antibody concentrations ≥ 12 IU/L, most participants had an anamnestic response to a challenge dose which indicates immune memory and likely protection. There is a need for more studies in this area, and also booster vaccinations may be more applicable in high prevalence settings and or among groups at higher risk of infection, for example household contacts of people living with hepatitis B.

Our results are subject to limitations. Importantly, for the observational KiGGS Wave 2 study anti-HBs antibodies were measured at the time of study participation, therefore the time since vaccination varied considerably. Furthermore, we did not measure an acute response to vaccination, but rather a mixture of response and waning.

Moreover, only 2,489 of the 3,567 participants (69.8%) who took part in the examination arm of the KiGGS Wave 2 study were included in our analyses and we found some differences in age and geographical place of living between those included versus those excluded, however the weighted analyses accounted for this difference in the probability of selection in the sample. One explanation for the lower mean age among those excluded may be that parents tended to be less willing to agree to a venous puncture when their children were of younger age. While there may be differences that we were unable to account for, implications of study participation have likely had limited impact on our results, as sociodemographic differences may affect access to (and uptake of) vaccines, but are unlikely to influence the biological response to vaccination. Fitting and comparing statistical models is normally done using the likelihood-ratio or AIC test, however this is challenging using survey data [44]. We assessed model fit using the Wald test for each added variable [44].

While there are important limitations to our study, there are also several strengths. The KiGGS Wave 2 data are based on a probability-based nation-wide sample, with a rigorous recruitment strategy and a high participation rate. Survey weights were included to account for the unequal probabilities of selection into the sample according to the national level regarding age, sex, federal state, migration background, and the education level of the parents.

In conclusion, we found that almost two thirds of the study participants had an anti-HBs level ≥ 10 IU/L at the time of the examination. We found that having a recommended schedule or three versus four doses did not have an influence on anti-HBs level. Future studies are needed to improve the understanding of the association between anti-HBs level and adequate and sustaining protection against HBV.

### Supplementary Information

**Supplementary Material 1.**

**Supplementary Material 2.**

**Supplementary Material 3.**

**Supplementary Material 4.**

## Data Availability

The data cannot be made publicly available because informed consent from participants did not cover the public deposition of data. However, the data underlying the analyses in this article are archived in the Epidemiological Data Centre of the Department 2: Epidemiology and Health Monitoring at the Robert Koch Institute in Berlin and can be accessed on site upon reasonable request (please contact corresponding author).
