# Supplementary material for: Prevalence of vaccine-derived hepatitis B surface antibodies in children and adolescents in Germany: results from a population-based survey, 2014–2017
Source: BMC Infect Dis. 2024 Mar 15;24:318. doi: 10.1186/s12879-024-09201-7 (PMC10941582; doi:10.1186/s12879-024-09201-7)
Supplement: Supplementary file 1 — Supplementary Material 1. [file 12879_2024_9201_MOESM1_ESM.docx]

# Additional file 1: Flowchart of participants included in the analyses

**n=329**
(Participants without or with unreadable vaccination book)

**n=3,567**
(Participants in examination part)

**n=73**(Participants with at least one missing date for an HBV vaccination)

**n=6**
(Anti-HBc and/or HBs-Ag positive participants (one not

vaccinated))

**n=464**
(participants without measurement of Anti-HBs)

**n=2,489**(Anti-HBc and HBs-Ag negative participants with ≥ 1 dose of active HBV vaccination and measurement of Anti-HBs )

**n=2,953**
(Anti-HBc and HBs-Ag negative participants with ≥ 1 dose of active HBV vaccination)

**n=2,959**
(Participants with ≥ 1 dose of active HBV vaccination)

**n=206**
(Participants with 0 doses of active HBV vaccination)

**n=3,165**
(Participants with vaccination books and date of HBV vaccination)

**n=3,238**(Participants with vaccination book or unvaccinated without vaccination book)
