# Supplementary material for: Prevalence of vaccine-derived hepatitis B surface antibodies in children and adolescents in Germany: results from a population-based survey, 2014–2017
Source: BMC Infect Dis. 2024 Mar 15;24:318. doi: 10.1186/s12879-024-09201-7 (PMC10941582; doi:10.1186/s12879-024-09201-7)
Supplement: Supplementary file 2 — Supplementary Material 2. [file 12879_2024_9201_MOESM2_ESM.docx]

# Additional file 2: Sociodemographic characteristics among population in analysis (n=2,489) and those excluded (n=1,078)

| **Variable** | **Included participants (n=2,489)** | **Excluded participants (n=1,078)** |  | ***X*^2^ (p value)** |
| --- | --- | --- | --- | --- |
| **Sex** |  |  |  |  |
| Male | 1,226 (49.3%) | 540 (50.1%) |  | 0.21 (0.647) |
| Female | 1,263 (50.7%) | 538 (49.9%) |  |  |
| **Total** | 2,489 (100%) | 1,078 (100%) |  |  |
| **Geographical place of living** |  |  |  |  |
| Living in Eastern Germany (incl. Berlin) | 893 (35.9%) | 336 (31.2%) |  | 7.39 (0.007) |
| Living in Western Germany | 1,596 (64.1%) | 742 (68.8%) |  |  |
| **Total** | 2,489 (100%) | 1,078 (100%) |  |  |
| **Age** |  |  |  |  |
| 3-6 years | 525 (21.1%) | 360 (33.4%) |  | 84.2 (0.000) |
| 7-10 years | 613 (24.6%) | 298 (27.6%) |  |  |
| 11-13 years | 619 (24.9%) | 197 (18.3%) |  |  |
| 14-17 years | 732 (29.4%) | 223 (20.7%) |  |  |
| **Total** | 2,489 (100%) | 1,078 (100%) |  |  |
