# Supplementary material for: Prevalence of vaccine-derived hepatitis B surface antibodies in children and adolescents in Germany: results from a population-based survey, 2014–2017
Source: BMC Infect Dis. 2024 Mar 15;24:318. doi: 10.1186/s12879-024-09201-7 (PMC10941582; doi:10.1186/s12879-024-09201-7)

# **Additional File 3: Time since last dose (in years) and anti-HBs level stratified per sex, age group and recommended schedule**

Figure 1: Time since last dose (in years) and anti-HBs level by sex (N=2,472*)


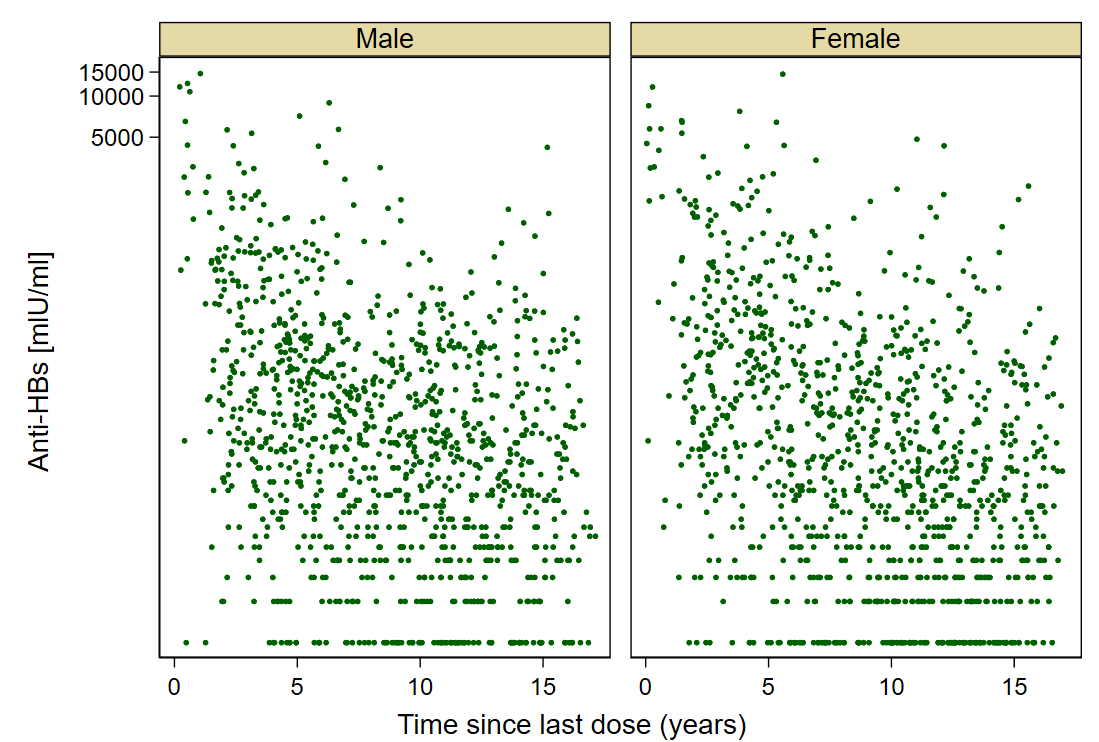


Figure 2: Time since last dose (in years) and anti-HBs level among males by age-group (N=2,472*)


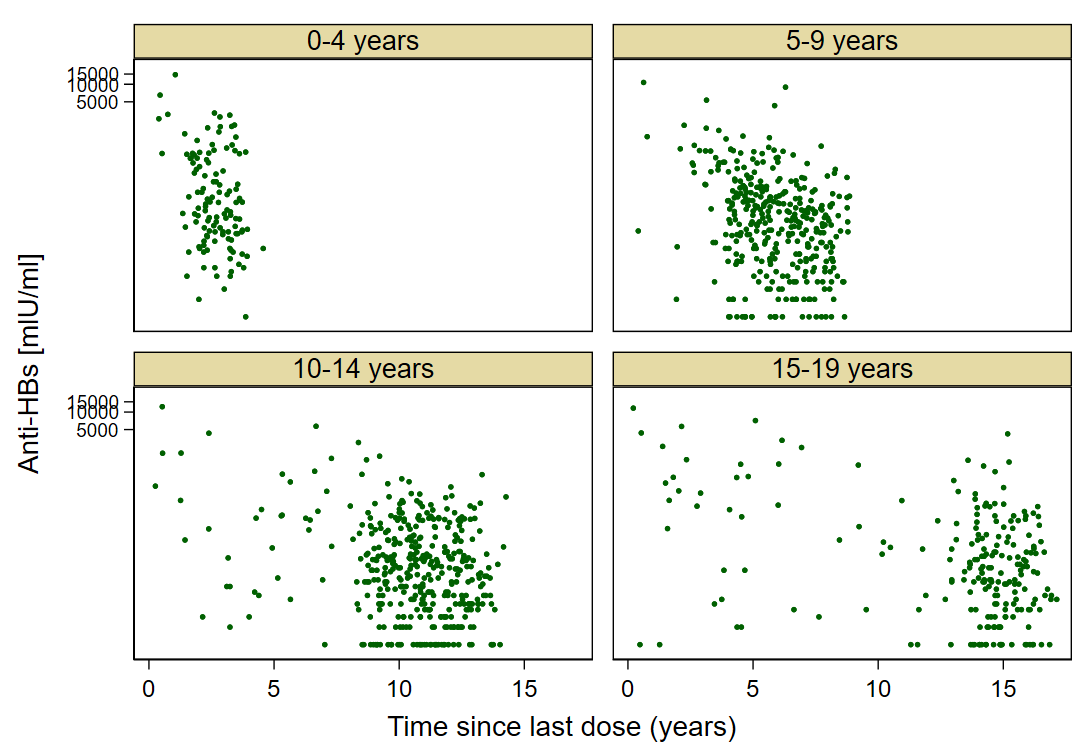


Figure 3: Time since last dose (in years) and anti-HBs level among females by age-group (N=2,472*)


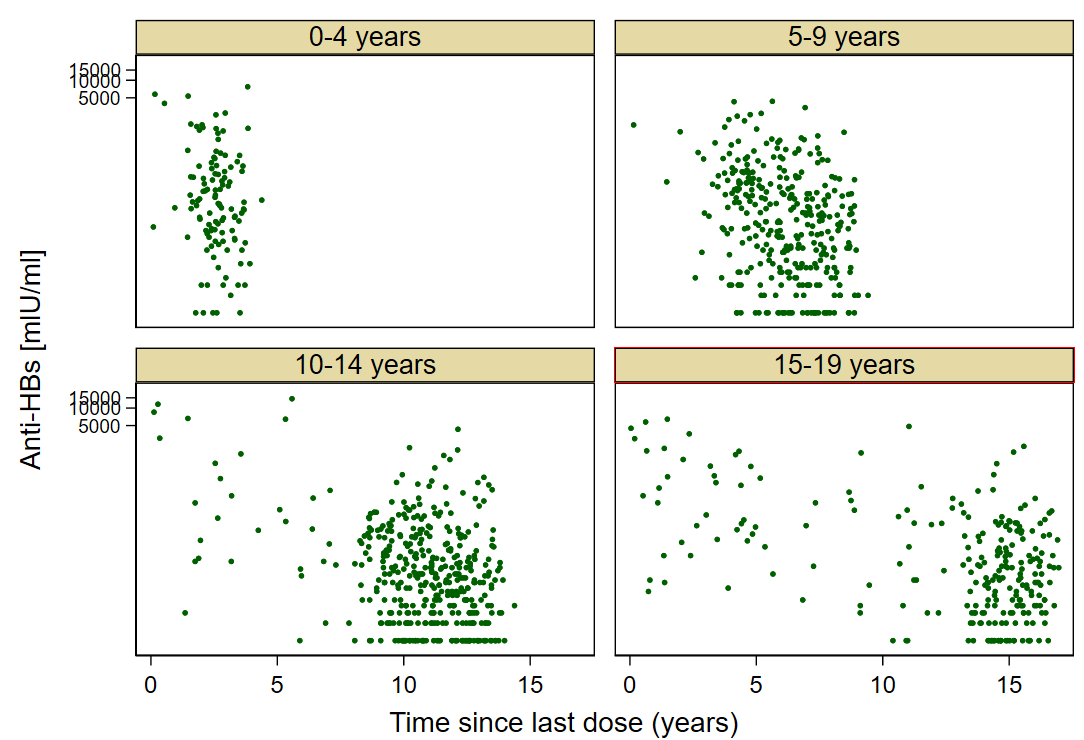


Figure 4: Time since last dose (in years) and anti-HBs level by recommended series (N=2,472*)


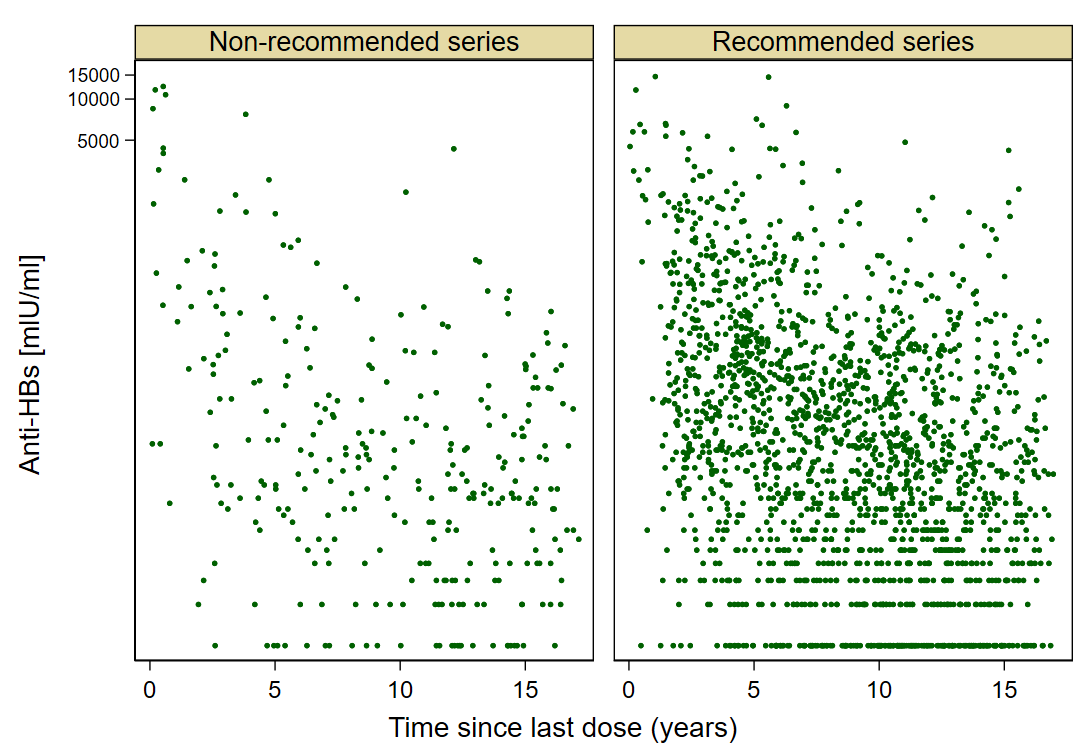


Figure 5: Time since last dose (in years) and anti-HBs level by three versus four doses (recommended series) (N=2,472*)


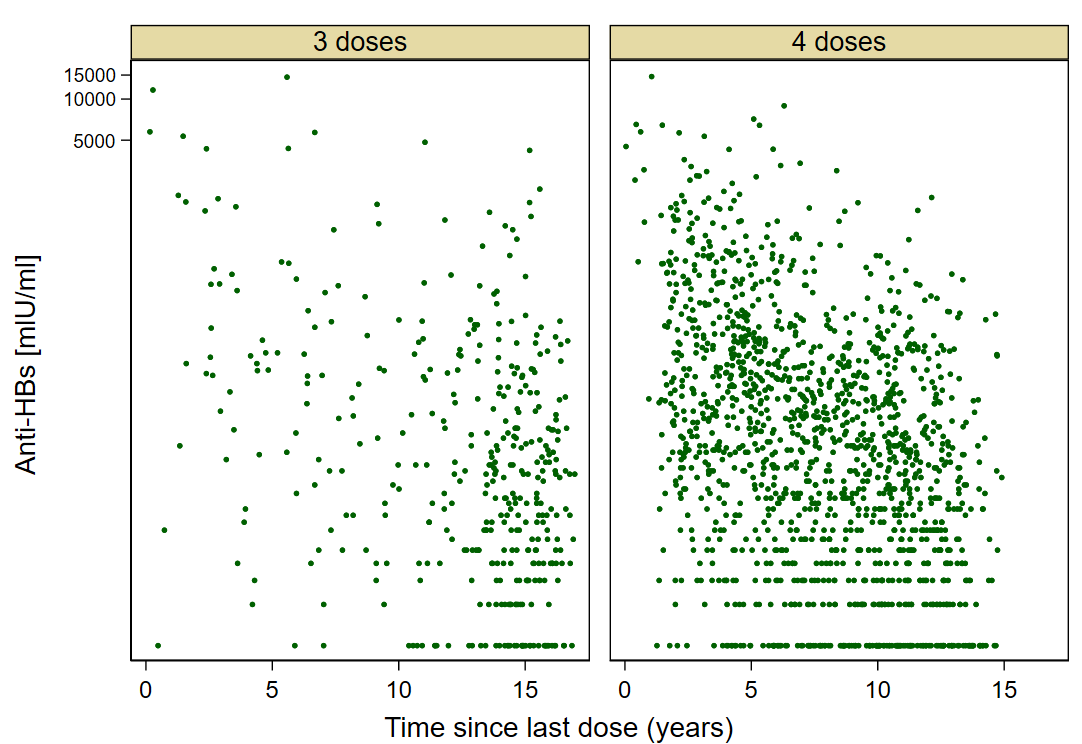


Figure 6: Time since last dose (in years) and anti-HBs level by monovalent and polyvalent vaccines (N=2,472*)


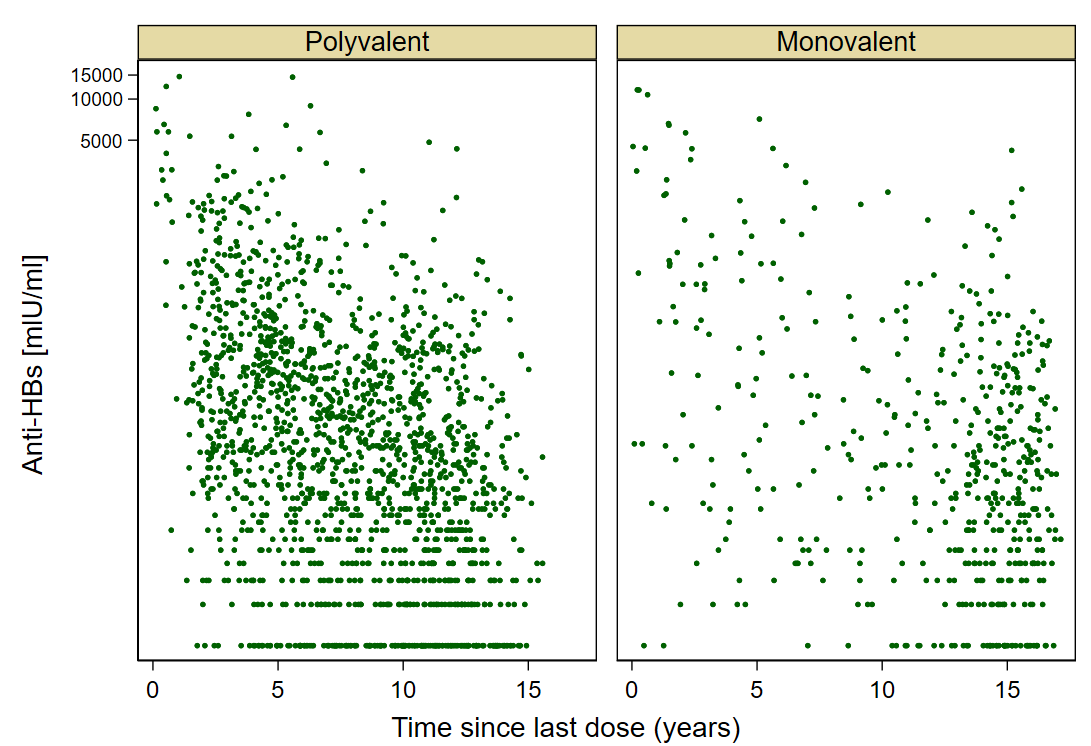

Supplement: Supplementary file 3 — Supplementary Material 3. [file 12879_2024_9201_MOESM3_ESM.docx]
